# The relationship between semen seminal plasma ions and sperm cell velocities of wild-caught longspine scraper, *Capoeta trutta*

**DOI:** 10.5194/aab-62-557-2019

**Published:** 2019-09-26

**Authors:** Mustafa Erkan Özgür, Zeynep Maraş, Selim Erdoğan

**Affiliations:** 1 Department of Aquaculture, Faculty of Fishery, Malata Turgut Özal University, 44210, Malatya, Turkey; 2 Department of Analytical Chemistry, Faculty of Pharmacy, İnönü University, 44280, Malatya, Turkey

## Abstract

In this study, semen seminal plasma contents and the motility of sperm cells were determined in *Capoeta trutta* via a computer-assisted sperm analysis system. In addition, we evaluated the relationship between semen seminal plasma ions and the velocities of sperm cells. Although the predominant ions were K (206.84±20.61 mg L${}^{-1}$) and Na (128.06±23.82 mg L${}^{-1}$) in the semen seminal plasma, Ca (14.05±4.13 mg L${}^{-1}$) and Mg (3.35±0.44 mg L${}^{-1}$) were not predominate according to our results. However, partially strong relationships between the curvilinear velocity value (VCL) and K ($R^{2}=0.67$; p<0.05) were found, while it was moderate with Mg ($R^{2}=0.48$; p<0.05). There was a weak relationship with Na ($R^{2}=0.17$; p<0.05) and Ca ($R^{2}=0.34$; p<0.05). In our results, while the trace metals were determined as Zn > Al > B > Li > Cu in semen seminal plasma, they are not correlated with sperm cell velocities. Finally, we hope that the present information on the motility parameters of *Capoeta trutta* in this paper will eventually help artificial insemination in reproduction practices.

## 1 Introduction

*Capoeta trutta* (Heckel, 1843) is a species in the Cyprinidae family. The fish species is distributed in the Euphrates and Tigris rivers in Turkey, Iran, Iraq, and Syria (Esmaeili et al., 2010; Demirsoy, 1993). *Capoeta trutta* has been listed as “least concern” on the IUCN Red List of Threatened Species since 2014, but its population has been decreasing in its natural habitat because of overfishing and water pollution (Freyhof, 2014).

The aquatic species should be domesticated because commercial aquaculture has developed very rapidly, and threatened populations have needed additional restoration in wild habitats (Lorenzen et al., 2012). However, the control of quality gametes is important for the performance of brood stocks in aquaculture systems. Therefore, the semen quality is very important for fish farming industry, commercial species, and biotechnological applications (Bobe and Labbé, 2010; Cabrita et al., 2014; Şahinöz et al., 2008). The motility, duration of motility and density, pH, spermatocrit, and seminal plasma contents are major parameters to determine the semen quality in fish (Billard and Cosson, 1992; Rurangwa et al., 2004). However, seminal plasma has very important ions that support the viability, motility, or immobilization of sperm cells. In teleost fish species, the sperm cells are immobilized in the testes and seminal fluid, and the start of motility depends on the conditions of the fertilization environment. The ion concentrations (${\mathrm{Na}}^{+}$, $K^{+}$, ${\mathrm{Ca}}^{2+}$, ${\mathrm{Mg}}^{2+}$, ${\mathrm{HCO}}_{3}^{-}$ etc.), osmolality, and pH in activator media are among the most important to regulate motility of sperm cells, in addition to other factors (Alavi and Cosson, 2006; Browne et al., 2015; Dzyuba and Cosson, 2014). For example, rainbow trout sperm cells were inactivated after being diluted with solutions containing high concentrations of the $K^{+}$ ion. However, the isotonic NaCl solution triggers immediate activation of sperm cell motility (Alavi and Cosson, 2006). For the motility of rainbow trout sperm cells, ${\mathrm{Na}}^{+}/H^{+}$ exchange may also be responsible for the participation of ${\mathrm{Na}}^{+}$ ions (Dzyuba and Cosson, 2014). However, researchers have found that intracellular Ca ion concentrations increase when the motility of sperm cells begins. Therefore, Ca ion concentration is a necessity for the motility of sperm cells (Alavi et al., 2011; Alavi and Cosson, 2006; Cosson et al., 1989; Tanimoto and Morisawa, 1988). However, some trace elements such as Zn, Mg, Cu, and Ca are important for the maintenance of normal spermatogenesis, sperm maturation, and DNA metabolism (Yuyan et al., 2008). There are some studies that associated trace elements with the parameters of oxidative stress and sperm cell motility in *Acipenser ruthenus* (Li et al., 2010); there are also associated relationships between trace elements and sperm cell motility in *Cyprinus carpio* (Kovacik et al., 2018), *Barbus grypus* (Ögretmen et al., 2014), the levels and effects of trace elements of semen seminal plasma in infertile men (Bassey et al., 2013; Vickram et al., 2013), and bovine semen (Tvrdá et al., 2013). Computer-assisted sperm analysis (CASA) systems are very popular for the practical analysis of sperm cell motility in various species and have combined the methods of predicting the analyzer's emotions with classical methods used in the past (Fauvel et al., 2010; Özgür et al., 2019).

The reproduction properties of *Capoeta trutta* can help to understand their management, conservation in nature, and reproduction status in artificial conditions. Thus, we studied some parameters of reproduction, including sperm cell motility, sperm cell density, and the contents of semen seminal plasma.

## 2 Material and methods

### 2.1 Semen sample collection

Wild males of *Capoeta trutta* (weight: 350±20 g, total length: 26±5.6 cm, N=10) were caught at Karakaya Dam Lake in the upper part of the Euphrates River, Malatya Province, Turkey, on May 2018. The semen samples were collected without hormone injection. The sexual maturity of the fish was confirmed by the urogenital opening and breeding tubercles on the nose. Therefore, semen samples collected in Eppendorf tubes with gentle pressure to the abdomen. Then, they were rapidly transported in Styrofoam boxes with ice to the laboratory and were analyzed. Care was taken to avoid any contamination by urine during the collection of semen samples.

### 2.2 The determination of sperm cell motility

Fresh semen samples from 10 individual fish were diluted at a ratio of 1:100 with immotile solution (IMS) (128.4 mM NaCl, 2.7 mM KCl, 1.4 mM ${\mathrm{CaCl}}_{2}$, 2.4 mM ${\mathrm{NaHCO}}_{3}$; pH 8.1) (Linhart et al., 2000) and were stored at 4 ${}^{\circ }$C until the analysis was performed, 1 h. Then, semen samples were activated under the microscope to determine the parameters of sperm cell motility at a ratio of 1:20 with activation solution (AS) (mM NaCl; 5 mM KCl; 20 mM Tris–HCl, pH 8) (Linhart et al., 2000) at room temperature. Sperm cells were analyzed without coverslips. All semen samples were kept on ice and examined under an Olympus CX31 microscope with 200× magnification lens and a Sony CCD camera with 30 fbs video recording capacity. The motility of sperm cells was analyzed via the computer-assisted sperm analysis system BASA-Sperm Aqua, produced by Merck Biotechnology Ltd. Co. in Turkey. The values of motility parameters such as VSL (straight line velocity, µm s${}^{-1}$), VCL (curvilinear velocity, µm s${}^{-1}$), VAP (angular path velocity, µm s${}^{-1}$), LIN (linearity, %, (VSL/VCL) × 100), BCF (beat cross frequency, Hz), ALH (amplitude of lateral displacement of the sperm cell head, µm), and MAD (mean angular displacement, (${}^{\circ }$)) (Fauvel et al., 2010; Özgür et al., 2019) are examined in the study. The following setting were used in the picture settings and parameters of BASA Sperm Aqua: acquired time delay of 0, an image field maximum of 60; images per record of 90, frame per second minimum of 30, track immotile level of 5, track motile level of 25, velocity maximum of 500.

The semen samples were diluted with 125 mM NaCl up to 16-fold and determined in a spectrophotometer with the absorbance at 505 nm for sperm cell concentration (Ciereszko and Dabrowski, 1993).

### 2.3 The analysis of semen seminal plasma ions

Semen seminal plasma was collected after centrifugation of the semen at 3400 g for 10 min in Beckman L-8-70M ultracentrifuge (Rotor SW-28, Munich, Germany). Seminal plasma was centrifuged twice to avoid possible contamination with sperm cell and stored at -20 ${}^{\circ }$C until analysis. The samples of semen were digested in the ultrasonic bath for 10 min by using 1 mL ${\mathrm{HNO}}_{3}$. Digested solutions were diluted with deionized water until adjusted to 10 mL. After dilution, they were analyzed for Na, K, Ca, Mg, Al, B, Cu, Li, and Zn (Massanyi et al., 2008; Özgür et al., 2015; Slivkova et al., 2009). Metal concentrations were determined by inductively coupled plasma optical emission spectrometry (ICP-OES VARIAN 725-ES, with CCD detector). All standard solutions (0.01, 0.05, 0.1, 0.2, 0.5, 1.0, 2.0, 5.0, 10.0, and 100 mg L${}^{-1}$) were prepared by diluting 1 mg mL${}^{-1}$ stock multielement standard solutions for ICP-OES. The optimum instrumental parameters of ICP-OES were, for example, radiofrequency power (1.2 kW), plasma gas flow rate (15.0 L min${}^{-1}$), auxiliary gas flow rate (1.5 L min${}^{-1}$), nebulizer flow rate (0.75 L min${}^{-1}$), replicate read time (3 s), delay (15 s), sample uptake delay (30 s), pump rate (15 rpm), rinse time (20 s), replicates (3), and torch (quartz for vertical view) for detection of multi-elements.

### 2.4 Statistical analysis

The relationships between all data were tested by using the bivariate correlation coefficients of Pearson. Then, linear and non-linear regression models were investigated between parameters. Descriptive analysis (means ± SD, p<0.05) and the normality test were performed between the data in the SPSS 17 program.

## 3 Results

Some motility parameters were evaluated characterizing velocities (VSL, VCL, and VAP) and movement styles (LIN, BCF, ALH and MAD) in sperm cells and semen seminal plasma contents of *Capoeta trutta* (Table 1). Sperm cell concentration ranged from 7.5 to 11.84 (mean ± SE: 9.44±1.72) × 10${}^{9}$ sperm cell mL${}^{-1}$. The velocities of sperm cells were found: the straight line velocity (VSL, 52.84±6.32 µm s${}^{-1}$), the curvilinear velocity (VCL, 103.86±22.67 µm s${}^{-1}$), and the angular path velocity (VAP, 66.71±11.02 µm s${}^{-1}$). In the semen seminal plasma ions K (206.84±20.61 mg L${}^{-1}$) and Na (128.06±23.82 mg L${}^{-1}$) were the predominant ions. Ca and Mg were measured to be 14.05±4.13 and 3.35±0.44 mg L${}^{-1}$, respectively.

**Table 1:** The motility parameters of sperm cells and semen seminal plasma contents in *Capoeta trutta*.

N=10	Mean ± SD	Minimum	Maximum
Motility parameters			
VSL (µm s${}^{-1}$)	52.84±6.32	43.17	63.99
VCL (µm s${}^{-1}$)	103.86±22.67	64.62	135.30
VAP (µm s${}^{-1}$)	66.71±11.02	43.42	79.67
LIN (%)	24.39±5.11	16.33	41.38
BCF (Hz)	9.21±1.90	6.17	11.34
ALH (µm)	37.69±7.49	23.15	49.31
MAD (${}^{\circ }$)	0.02±0.01	0.01	0.04
Seminal plasma contents			
Ca (mg L${}^{-1}$)	14.05±4.13	8.82	22.06
K (mg L${}^{-1}$)	206.84±20.61	151.36	265.00
Mg (mg L${}^{-1}$)	3.35±0.44	2.66	4.20
Na (mg L${}^{-1}$)	128.06±23.82	88.50	168.67
Na/K	0.63±0.13	0.38	0.82
Al (mg L${}^{-1}$)	5.57±0.77	4.68	6.78
B (mg L${}^{-1}$)	4.07±3.27	0.69	11.82
Cu (mg L${}^{-1}$)	0.42±0.06	0.36	0.54
Li (mg L${}^{-1}$)	1.31±0.17	1.24	1.77
Zn (mg L${}^{-1}$)	7.37±0.92	6.90	9.80

**Figure 1:**
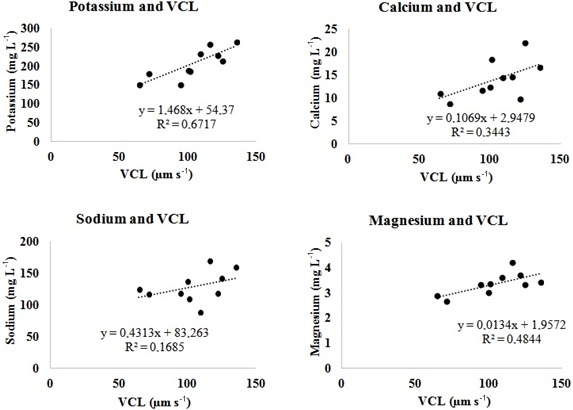
Linear regression graphics between some ions of semen seminal plasma and the VCL value of sperm cell in *Capoeta trutta*.

Positive relationships were found between K, Na, Ca, and Mg in semen seminal plasma and the values of VSL, VCL, and VAP in sperm cell velocities. However, the partially strong relationship with the VCL value was with K ($R^{2}=0.67$; p<0.05), while it was moderate with Mg ($R^{2}=0.48$; p<0.05) and comparable with Na ($R^{2}=0.17$; p<0.05) or Ca ($R^{2}=0.34$; p<0.05) (Fig. 1, Table 3). The correlation between the contents of semen seminal plasma and motility parameters of sperm cells is shown in Table 2. According to our results, K and Mg ions correlated positively with the VCL value of sperm cells, r=0.820 and r=0.696, p<0.01, respectively. In this study, there was no correlation between the trace metals and sperm cell velocities according to our results (Table 2).

**Table 2:** Pearson correlations between sperm cell motility and semen seminal plasma contents of *Capoeta trutta*.

	Pearson correlations														
	VSL	VCL	VAP	LIN	BCF	ALH	MAD	Ca	K	Mg	Na	Al	B	Cu	Li
VCL	0.555														
VAP	0.755${}^{a}$	0.504													
LIN	-0.357	-0.580	-0.154												
BCF	0.130	0.574	0.023	-0.371											
ALH	0.021	-0.131	0.223	0.546	-0.349										
MAD	-0.390	-0.803${}^{b}$	-0.183	0.695${}^{a}$	-0.231	0.299									
Ca	0.242	0.587	0.009	-0.590	0.394	-0.537	-0.575								
K	0.451	0.820${}^{b}$	0.362	-0.496	0.375	0.004	-0.823${}^{b}$	0.361							
Mg	0.324	0.696${}^{a}$	0.452	-0.605	0.244	0.221	-0.609	0.305	0.716${}^{a}$						
Na	-0.313	0.410	-0.135	0.043	0.204	-0.002	-0.471	0.279	0.459	0.321					
Al	0.268	0.423	0.355	-0.644${}^{a}$	0.056	0.103	-0.537	-0.063	0.608	0.820${}^{b}$	0.075				
B	-0.131	0.047	0.059	0.131	-0.345	-0.356	-0.198	0.278	-0.062	-0.296	0.319	-0.365			
Cu	0.061	0.108	0.398	-0.310	-0.187	-0.294	-0.199	-0.214	0.007	0.133	-0.057	0.438	0.381		
Li	-0.005	0.342	0.170	-0.377	0.369	0.075	-0.253	-0.328	0.337	0.468	0.021	0.691${}^{a}$	-0.385	0.508	
Zn	-0.007	0.354	0.208	-0.419	0.322	0.067	-0.288	-0.305	0.373	0.532	0.053	0.756${}^{a}$	-0.348	0.558	0.992${}^{b}$

${}^{a}$ Correlation is significant at the 0.05 level (two-tailed). ${}^{b}$ Correlation is significant at the 0.01 level (two-tailed).

**Table 3:** Linear regression relationship between some ions of semen seminal plasma and the velocities of sperm cells in *Capoeta trutta*.

Parameters	Regression equation	Regression square
Potassium–VSL	y=2.90x+53.59	$R^{2}$ = 0.20
Potassium–VAP	y=1.35x+117.72	$R^{2}$ = 0.13
Calcium–VSL	y=0.16x+5.67	$R^{2}$ = 0.06
Calcium–VAP	y=0.00x+13.83	$R^{2}$ = 0.00
Sodium–VSL	y=-1.18x+190.46	$R^{2}$ = 0.10
Sodium–VAP	y=-0.29x+147.58	$R^{2}$ = 0.02
Magnesium–VSL	y=0.02x+2.17	$R^{2}$ = 0.11
Magnesium–VAP	y=0.02x+2.15	$R^{2}$ = 0.20

## 4 Discussion

The assessment semen quality can give great information for gamete management and the protocols of artificial reproduction of wild fish species in captivity (Alavi et al., 2006; Mylonas et al., 2010). Therefore, we aimed to determine gamete quality parameters on a domestic fish species, *Capoeta trutta* in the Euphrates River. In this study, we analyzed the relationships between sperm cell velocities and semen seminal plasma contents in *Capoeta trutta*. Thereby, the values of VSL, VCL, and VAP were higher than those for *Barbus sharpeyi*, *Catla catla*, and *Labeo rohita* (Kalbassi et al., 2013); they were similar to *Labeo calbasu, Cirrhinus mrigala*, *Hypophthalmichthys molitrix*, and *Ctenopharyngodon idella* (Verma et al., 2009). However, the sperm cell density of *Capoeta trutta* ($9.44\times 10^{9}$) was found to be more than the sperm cell density of *Liza abu* ($4.27\times 10^{9}$) (Şahinöz et al., 2008) and *Capoeta damascina* ($∼5\times 10^{9}$) (Zadmajid et al., 2018), while it was found to be less for *Barbus grypus* ($18.8\times 10^{9}$) (Khodadadi et al., 2016), *Cyprinus carpio* (16.96–$18.81\times 10^{9}$) (Cejko et al., 2014), and *Ctenopharyngodon idella* ($15.43\times 10^{9}$) (Bozkurt et al., 2008). These differences can be caused by hormonal treatments which increase sperm cell density in some fish species (Zadmajid et al., 2018).

There are very important ions (${\mathrm{Na}}^{+}$, $K^{+}$, ${\mathrm{Ca}}^{2+}$, ${\mathrm{Mg}}^{2+}$, ${\mathrm{HCO}}_{3}^{-}$ etc.) in semen seminal plasma which support the viability, motility, or immobilization of sperm cells which are immobilized in the testes and semen seminal plasma in teleost fish species. The initial motility depends on the conditions of the fertilization environment (Alavi and Cosson, 2006; Browne et al., 2015; Dzyuba and Cosson, 2014). For example, the motility of rainbow trout sperm cells was inhibited with high concentrations of the $K^{+}$ ion (Alavi and Cosson, 2006). However, there is a positive correlation between sperm cell velocity and Ca ion content of semen seminal plasma (Alavi et al., 2011; Dziewulska and Domagała, 2013; Nynca et al., 2014). On the other hand, $K^{+}$ ions completely inhibited sperm cell activation (Alavi et al., 2011; Ögretmen et al., 2014).

In our study, ${\mathrm{Na}}^{+}$ and $K^{+}$ were seen as the main electrolytes involved in the maintenance of the osmolality of semen seminal plasma (Alavi and Cosson, 2006; Morisawa et al., 1983; Zadmajid, 2016). However, a positive correlation has been reported among osmolality, ${\mathrm{Na}}^{+}$, and ${\mathrm{Cl}}^{-}$ in the seminal plasma of *Alburnus alburnus* (Lahnsteiner et al., 1996). This status can occur due to the different species (Alavi and Cosson, 2006; Verma et al., 2009).

Positive linear regression relationships (weak in VSL and VAP in Table 3, moderately strong in VCL in Fig. 1) were found between velocities and ${\mathrm{Na}}^{+}$, $K^{+}$, Mg, and ${\mathrm{Ca}}^{2+}$ in the semen seminal plasma of *Capoeta trutta*. In some teleosts, some researchers have found a positive relationship between sperm cell motility and $K^{+}$ and ${\mathrm{Na}}^{+}$ of seminal plasma (Alavi and Cosson, 2006; Bozkurt et al., 2008; Lahnsteiner et al., 1996) and ${\mathrm{Ca}}^{2+}$ of northern pike *Esox lucius* (Siddique et al., 2016a). However, the ratio of ${\mathrm{Na}}^{+}/K^{+}$ in the semen seminal plasma is vital in understanding the characteristics of fish sperm cell motility (Alavi and Cosson, 2006). The ratio of ${\mathrm{Na}}^{+}/K^{+}$ in the semen seminal plasma of *Capoeta trutta* was determined to be about 1:2, and it was lower than that for northern pike (Siddique et al., 2016b), some species of Salmonidae and Acipenseridae (Alavi and Cosson, 2006), and *Barbus grypus* (Khodadadi et al., 2016). However, it was parallel with *Barbus sharpeyi* (Alavi et al., 2010), *Ctenopharyngodon idella* (Bozkurt et al., 2008), and *Cyprinus carpio* (Bozkurt et al., 2009). These differences were probably present due to the individual characteristics of the species (Verma et al., 2009).

In conclusion, sperm cells generally move on a straight or slightly curved route after activation, which was found to be similar to and/or closely comparable to the results of some authors according to the obtained parameters (Alavi et al., 2014; Fitzpatrick et al., 2008). Trace metals such as Zn, Al, B, Li, and Cu in semen seminal plasma were not correlated with sperm cell velocities. A similar status was confirmed for Zn in human semen (Bassey et al., 2013), while this was not the case for Zn in common carp semen (Kovacik et al., 2018) and human semen (Vickram et al., 2013), and for Zn and Cu in bovine semen (Tvrdá et al., 2013). These differences are probably due to the individual characteristics of the species (Verma et al., 2009), the activity of male reproductive accessory organs (Alavi et al., 2004), and the metal pollution of water (Acosta et al., 2016).

Finally, our results have shown that seminal plasma ions such as ${\mathrm{Na}}^{+}$, $K^{+}$, Mg, and ${\mathrm{Ca}}^{2+}$ were correlated with sperm cell velocities, while there is only the partially strong relationship between the VCL value and $K^{+}$ ion of sperm seminal plasma. However, we are hopeful that our data can lead to a better understanding of fertilization mechanisms of *Capoeta trutta* sperm cells because the knowledge of sperm cell motility and semen seminal plasma contents is a prerequisite for the successful assessment of the reproductive capacity of wild fish species.

## Data Availability

The data used in the present study are confidential and therefore not publicly available.

## Appendix A

**Table:**

Abbreviations and their definitions	
VSL	the straight line velocity of sperm cell
VCL	the curvilinear velocity of sperm cell
VAP	the angular path velocity of sperm cell
LIN	the linearity of sperm cell
BCF	the beat cross frequency of sperm cell
ALH	the amplitude of lateral displacement of the sperm cell head
MAD	the mean angular displacement of sperm cell
Ca	calcium
K	potassium
Mg	magnesium
Na	sodium
Al	aluminum
B	boron
Cu	copper
Li	lithium
Zn	zinc
